# Reduced FcRn-mediated transcytosis of IgG2 due to a missing Glycine in its lower hinge

**DOI:** 10.1038/s41598-019-40731-2

**Published:** 2019-05-14

**Authors:** Nigel M. Stapleton, Maximilian Brinkhaus, Kathryn L. Armour, Arthur E. H. Bentlage, Steven W. de Taeye, A. Robin Temming, Juk Yee Mok, Giso Brasser, Marielle Maas, Wim J. E. van Esch, Mike R. Clark, Lorna M. Williamson, C. Ellen van der Schoot, Gestur Vidarsson

**Affiliations:** 1 Sanquin Research, Department of Experimental Immunohematology, Amsterdam, The Netherlands, and Landsteiner Laboratory, Amsterdam UMC, University of Amsterdam, Amsterdam, The Netherlands, Plesmanlaan 125, Amsterdam, 1066 CX The Netherlands; 2 Present Address: HALIX B.V., J.H. Oortweg 15/17, 2333 CH Leiden, The Netherlands; 3 Department of Pathology, Division of Immunology, University of Cambridge, Tennis Court Road, Cambridge, CB2 1QP UK; 4 Department of Haematology, University of Cambridge, Cambridge, UK; 5 NHS Blood and Transplant, Long Road, Cambridge, CB2 2PT UK; 6 Present Address: LifeArc, Open Innovation Campus, Stevenage, SG1 2FX UK; 7 Present Address: Clark Antibodies Ltd, 10 Wellington Street, Cambridge, CB1 1HW UK; 8 Sanquin Reagents, Amsterdam, Netherlands

**Keywords:** Antibody generation, Antibodies

## Abstract

Neonatal Fc-receptor (FcRn), the major histocompatibility complex (MHC) class I-like Fc-receptor, transports immunoglobuline G (IgG) across cell layers, extending IgG half-life in circulation and providing newborns with humoral immunity. IgG1 and IgG2 have similar half-lives, yet IgG2 displays lower foetal than maternal concentration at term, despite all known FcRn binding residues being preserved between IgG1 and IgG2. We investigated FcRn mediated transcytosis of V_H_-matched IgG1 and IgG2 and mutated variants thereof lacking Fc-gamma receptor (FcγR) binding in human cells expressing FcRn. We observed that FcγR binding was not required for transport and that FcRn transported less IgG2 than IgG1. Transport of IgG1 with a shortened lower hinge (ΔGly236, absent in germline IgG2), was reduced to levels equivalent to IgG2. Conversely, transport of IgG2 + Gly236 was increased to IgG1 levels. Gly236 is not a contact residue between IgG and FcRn, suggesting that its absence leads to an altered conformation of IgG, possibly due to a less flexible Fab, positioned closer to the Fc portion. This may sterically hinder FcRn binding and transport. We conclude that the lack of Gly236 is sufficient to explain the reduced FcRn-mediated IgG2 transcytosis and accounts for the low maternal/fetal IgG2 ratio at term.

## Introduction

During the first months after birth, before infants acquire their own immunity, they are protected by maternal IgG class antibodies, transferred by the neonatal Fc-receptor (FcRn)-mediated transplacental transcytosis^1–5^. Critically for this transport, FcRn binds IgG with nanomolar affinities at low pH (≤6.5) (found in intracellular vacuoles), while at physiological pH (7.4) this affinity is low^6–9^.

Once it is pinocytosed, the IgG containing vesicle acidifies, causing IgG present to bind to FcRn. Endocytosis and sorting signals in the cytoplasmic tail of FcRn cause FcRn-IgG complexes to be routed away from the lysosomal pathway to the plasma membrane^10–16^. Upon fusion with the plasma membrane in a series of short events^17^, the pH returns to physiological levels, IgG-FcRn complexes dissociate, IgG diffuses away and FcRn restarts its cycle. The net result, depending on the route of exocytosis, is either IgG-recycling or transcytosis^17–19^.

In human transplacental transport, IgG is generally thought to be transported from the maternal to fetal circulation in three steps: First IgG is pinocytosed by FcRn expressing syncytiotrophoblasts, it transverses the villus interstitium passively by bulk flow and finally IgG is transported across fetal villus endothelial cells^20^. During the first half of pregnancy, the IgG subclass composition in cord blood resembles that in maternal serum, although the total IgG concentration in cord blood remains lower. At term however the IgG1 concentration in cord blood significantly exceeds that found in maternal serum, with slightly less IgG4 being transported. The trans-placental transport of IgG2 and IgG3 is the least efficient, being roughly equal.^21–26^.

Interestingly, both IgG1 and IgG2 are reported to have similar fractional catabolic rates and half-lives of 21–28 days^27,28^, indicating that FcRn is able to rescue IgG1 and IgG2 equally efficiently from lysosomal degradation and implying that the mechanisms for transplacental transport may differ from those involved in apical recycling. In contrast, the half-life of IgG3 is approximately 7 days (comparable to that of other non-FcRn binding serum proteins), suggesting the FcRn rescuing function to be deficient for IgG3^27^. Despite this, IgG3 is transported across the placenta to a similar extent as IgG2, albeit to a lower degree than IgG1^21–26^. We recently demonstrated that the short half-life and lowered FcRn-meditated transcytosis of IgG3 were due to competition between different subclasses for FcRn-mediated rescue and that IgG3 was less successful due to a single amino acid difference within its FcRn-binding site. At this position (435) IgG3 has an arginine compared to histidine in other subclasses^28,29^. Individuals with the histidine-containing allotype of IgG3 (G3m(s,t) allotype which is uncommon in Europe, but relatively common in Asia and Africa), have half-life and transport across the placenta comparable to IgG1^25,28,30^.

Conversely, IgG1 and IgG2 are not known to differ in their affinities for FcRn or known IgG-FcRn contact residues^6,31^. Other FcγR might be involved in trafficking IgG across the placenta such as FcγRIIb^20^ and might favour certain subclasses. However, whereas FcRn is required, the role of FcγRIIb has been excluded in the trans-placental transport of mice^32,33^. Recently, we also found no evidence for any preference for either light chain isotype or IgG2 hinge isomer (formed by different disulphide-bridge formations between cysteines in the upper IgG2-hinge and the light chains), indicating that the difference in hinge flexibility reported by Dillon *et al*.^34^ does not affect FcRn function^26^. We have also recently generated a IgG variant lacking Fc-receptor effector functions by engrafting IgG2- and IgG4-derived amino acids onto IgG1 (Δnab^35^), which still binds FcRn. However, this variant showed surprisingly low FcRn-mediated transport^36^, warranting further investigation. Here we present a study on FcRn mediated IgG1 and IgG2 transcytosis and describe new findings on the differential transport of these subclasses. Our data suggest that IgG2 is transported less efficiently by FcRn due to the shorter and less flexible hinge of IgG2 than IgG1 and IgG3^34,37^. We postulate that the shortened hinge may influence the interaction of IgG2 with FcRn dimers because of steric hindrance with the plasma membrane^6,10^.

## Materials and Methods

### Cell culture

Human choriocarcinoma cells (JAR, American Type Culture Collection, Manassas, VA) were grown in Iscove’s Modified Dulbecco’s Medium (IMDM) (Cambrex, Verviers, Belgium), and melanoma cells (wild type A375 lacking FcRn expression (but expressing Beta-2-microglobuline (β2m)) and A375 transfected with the FcRn α-chain)^28^, in Roswell Park Memorial Institute (RPMI) 1640 medium (Invitrogen/Gibco, Carlsbad, California), both supplemented with L-glutamin (300 μg/ml, Invitrogen/Gibco), penicillin (100 U/ml, PAA Laboratories GmbH, Pasching, Germany), streptomycin (100 μg/ml, PAA) and 10% foetal calf serum (FCS, Bodinco, Alkmaar, The Netherlands). All cultures were carried out at 37 °C, in saturated humidity and 5% CO_2_ in air.

### Isolation of White Blood Cells (WBCs)

WBCs were isolated from 9 mL of freshly drawn blood, taken up in VACUETTE heparin blood collection tubes (Greiner Bio-one, Alphen a/d Rijn Nederland). Whole blood was separated by centrifugation at 1600 x g for 10 min, serum fraction was discarded. Red blood cells were lysed by three consecutive steps of hypotonic lysis in cell culture grade water (Gibco) for 30 sec, followed by addition of filtered (0.2 µm, Whatman) 10 x PBS. The protocol was performed at 4 °C/on ice.

### Fluorescence activated cell sorter (FACS)

A375 (wild-type and human FcRn) and JAR cells were treated with trypsin Ethylenediaminetetraacetic acid (EDTA) (0.5% and 0.2% (m/V)) for 5 min at culture conditions. 2 × 10^5^ cells per well were added to 96 well V-bottom plate (Costar/Corning) and stained with biotinylated mouse anti human Cluster of differentiation (CD)64 (FcγRI) (BD Pharmingen), CD32 (FcγRII) (Bio-Rad) and CD16 (FcγRIII) (SouthernBiotech, Birmingham, Alabama) for 30 min at 4 °C/on ice. Cells were washed thoroughly and incubated with Alexa Fluor 633 (AF 633) conjugated Streptavidin (Thermo Scientific). FACS analysis was performed on LSR-II (BD Biosciences), data was analyzed using FlowJo v10 (FlowJo, LLC). The experiment was performed in duplicate.

### IgG

For an overview of the antibodies used in this article and the figures they were used for, see Table 1.

**Table 1:** Nomenclature and mutations of antibodies used in this paper.

Name	Subclass	mutations in the backbone	specificity of CDR	Used in figure
B2G1	IgG1	none	HPA-1	2
B2G1Δnab	IgG1	K214T, D356E, L358M, A327G, A330S, P331S, E233P, L234V, L235A, deletion of G236	HPA-1	2
B2G1Δnac	IgG1	K214T, D356E, L358M, A327G, A330S, P331S, E233P, L234V, L235A	HPA-1	2
B2G2	IgG2	none	HPA-1	2
GDob1 IgG1	IgG1	none	*Streptococcus pneumoniae* serotype 6	3, 5, 7
GDob1 IgG1H435A	IgG1	H435A	*Streptococcus pneumoniae* serotype 6	3
GDob1 IgG1ΔG236	IgG1	deletion of Gly 236	*Streptococcus pneumoniae* serotype 6	3, 5, 7
GDob1 IgG2	IgG2	none	*Streptococcus pneumoniae* serotype 6	3, 5, 7
GDob1 IgG2H435A	IgG2	H435A	*Streptococcus pneumoniae* serotype 6	3
GDob1 IgG2 + G236	IgG2	insertion of Gly 236	*Streptococcus pneumoniae* serotype 6	3, 5, 7

A legend to the antibodies used in this paper, including the name, subclass, mutations, antigen specificity and list of figures in which they were used.

Recombinant B2G antibodies directed against human platelet antigen (HPA)-1a were produced as previously described^38^. For the B2G mutants, residues in IgG1 were substituted with corresponding amino acids from IgG2 and IgG4, reported to be responsible for their low affinity for FcγR. The Δc signifies alterations of the IgG1 backbone originating from IgG2 (E233P, L234V, L235A). Δb indicates the same substitution but accompanied by deletion of G236, a residue lacking in IgG2. Δa mutations originate from IgG4 (A327G, A330S, P331S). After alteration of three more residues aimed at removing allotypic variation from IgG1 (Δn, K214T, D356E, and L358M), this resulted in two variants of IgG1: Δnab and Δnac differing only by absence or presence of G236, both demonstrating severely reduced binding to FcγR but retaining all residues described to be involved in binding to FcRn^35,39,40^. Production of the wild type B2G and its variants were performed as described previously.

Recombinant V-gene matched IgG1 and IgG2 GDob1 antibodies, directed against *Streptococcus pneumoniae* serotype 6^41,42^, were produced in 293Freestyle cells (Invitrogen) according to the manufacturer’s instructions. GDob1IgG1H435A was generated as described^42^. Likewise, GDob1IgG1Δ236 G, GDob1IgG2 + 236 G, and GDob1IgG2H435A, were generated using the Quickchange Site-directed-mutagenesis kit (Stratagene, California, USA) using the following primers and their complementary primers:

GDob1IgG1Δ236G :5′ CCT CAG CAC CTG AAC TCC TGG G**^^ ^**AC CGT CAG TCT TCC TCC TCT TC 3′

GDob1IgG2 + 236G :5′ CTC TTC CTC AGC ACC ACC TGT GGC AGG A**GG G**CC GTC AGT CTT CCT CTT CCC CCC 3′

GDob1IgG2H435A :5′ GAG GCT CTG CAC AAC **GC**C TAC ACG CAG AAG AGC C 3′.

All mutations were confirmed by sequencing (ABI 373 Stretch automated sequencing machine, Applied Biosystems, Foster City, CA) prior to expression.

### Surface plasmon resonance (SPR)

Human FcRn was produced in-house as described in^43^ and^44^, respectively. Affinity measurements were performed with SPR using the IBIS MX96 (IBIS Technologies, Enschede, the Netherlands) as described by Dekkers *et al*.^45^.

Human FcRn was spotted in six duplicates with a three-fold dilution ranging from 30 nM to 1 nM on a SensEye G-streptavadin sensor (Ssens, Enschede, the Netherlands) using a Continuous Flow Microspotter (Wasatch Microfuidics, Salt Lake City, Utah)…

The GDob1 antibodies were injected in a two-fold dilution series starting from 0.49 nM to 125 nM over the FcRn–sensor in PBS-Tween 80 (0.075%) at pH 6.0. After every injection a two-step regeneration was performed using PBS pH8.5 + 0.075% Tween-80.

Calculation of the dissociation constant (KD) was done using an equilibrium analysis by intrapolation to Rmax = 1000 for FcRn^45^. Analysis and calculation of all binding data were carried out with Scrubber software version 2 (Biologic Software, Campbell, ACT, Australia) and Microsoft Office Excel 2013.

### IgG transcytosis

Transcytosis experiments with A375 (wild-type and human FcRn) and JAR cells were performed as previously described^28^. Briefly, 12 mm polycarbonate Transwell filters (0.4 μm pore size, Costar/Corning) were inoculated with 5 × 10^5^ cells, grown overnight to confluence, washed with phosphate buffered saline (PBS) and medium replaced with fresh medium (IMDM at pH 7.4 with supplements as stated above). Mixtures of IgG contained streptavidin-horseradish peroxidase (HRP) (Sanquin) to assess background transport. Apical to basolateral transport was calculated according to ([IgG]basolateral × 1.5 ml)/([IgG]input × 0.5 ml) × 100%. All experiments were performed in triplicate.

### Serum samples

Serum samples from the umbilical cord of three newborns and matched serum samples from mothers were drawn at birth. IgG subclass levels in these samples were determined by nephelometry as described below. Signed informed consent was obtained from all women, and the collection of blood samples and clinical data received approval by the Ethics Committee of the Leiden University Medical Centre (P02–200) as has been previously reported^24^, in accordance with relevant guidelines.

### IgG quantification

IgG subclass concentrations in serum samples were determined by Nephelometry (Behringer Nephelometer II, Behringer diagnostics, Deerfield, Illinois, USA) according to manufacturer’s protocols. In all *in vitro* studies, IgG subclasses were quantified by sandwich enzyme-linked immunosorbent assay (ELISA) using subclass specific mouse monoclonal antibodies (IgG1: MH161-1; IgG2: HP6062, Sanquin) to capture and a directly HRP conjugated monoclonal mouse anti-IgG (JDC-10, Southern Biotech, Birmingham, AL, USA) for detection. Conversion of 3,3′,5,5′-Tetramethylbenzidine (TMB) was used to quantitate HRP activity per well and absorptions were read using a Sunrise TECAN spectrophotometer. The concentrations were read from a standard curve made from the IgG preparations used for transport (*in vitro* studies) or IVIg.

### High-Pressure Liquid Chromatography Size-exclusion chromatography (HPLC-SEC)

ÄKTA explorer P900 (GE Healthcare) equipped with Superdex 200 10/300GL (GE Healthcare) was employed for HPLC-SEC. The column was adjusted to previously degassed PBS with two column volumes prior to sample run. 100 µL of purified Ab at a concentration of 1 mg/mL was injected manually. Analysis was performed at a flow of 0.5 mL/min, detecting at UV 280 nm.

### Statistical analysis and data sets

All data represent the mean and standard deviation of at least three independent experiments. All transcytosis assays consisted of three replicates. GraphPad Prism for Windows (GraphPad Software) was used for all statistical analysis. Significance was set at P < 0.05, and is indicated on all figures as *p ≤ 0.05; **p ≤ 0.01; ***p ≤ 0.001.

### “Informed Consent”

Signed informed consent was obtained from all women who donated serum sample of themselves and cord blood of their new-borns. The collection of blood samples and clinical data received approval by the Ethics Committee of the Leiden University Medical Centre (P02–200).

## Results

### IgG2 and IgG3 are transported across the placenta to a lesser extent than IgG1 and IgG4

**Figure 1:**
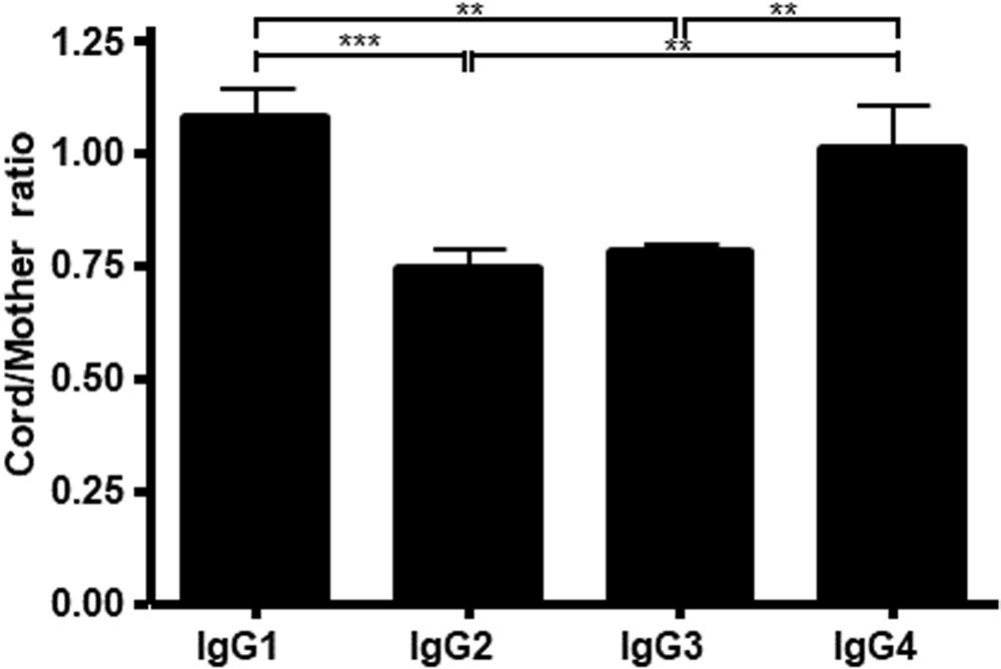
Relative IgG subclass concentrations in cord blood and maternal serum. The relative subclass concentration in cord blood as compared to the concentrations found in maternal serum, expressed as average cord/mother ratios for each IgG subclass. Data are from three paired mother – child samples and expressed as means plus standard deviation. All data are from 3 independent experiments, expressed as mean plus standard deviation. Data was analysed by one-way ANOVA with Tukey’s multiple comparison test and significance is shown as previously indicated.

We analyzed three matched mother and cord sera at term. Similar to previous studies^21,23–26^, we found that only IgG1 had a higher concentration in cord blood than in maternal serum, IgG2 and IgG3 were present in lower amounts in cord blood than in maternal serum, and IgG4 concentrations were equal (Fig. 1).

### FcRn mediated apical to basolateral IgG1 transport is more efficient than IgG2 transport *in vitro*

**Figure 2:**
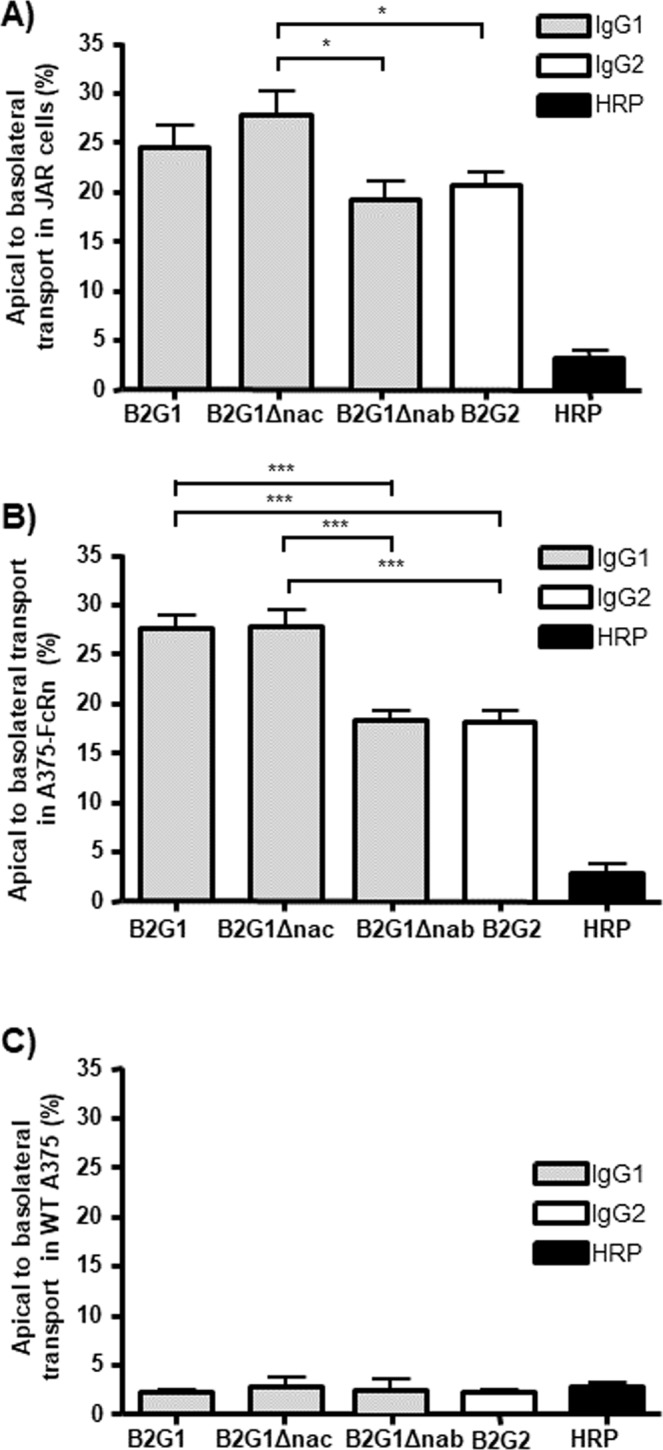
FcRn-mediated IgG transport is independent of FcγR and is influenced by the presence or absence of G236. Transport of IgG1 variants B2G1, B2G1Δnac and B2G1Δnab as well as IgG2 B2G2 was tested using different cell lines. (**A**) placenta derived cells (JAR). (**B**) A375-FcRn cells, transfected with the FcRn alpha chain. (**C**) 375 wild type cells lacking functional FcRn expression. All experiments were run for two hours and transport was from the apical to the basolateral side of a monolayer of cells. HRP was included as a measure of aspecific leakage and measured in the same samples as IgG. All data are from 3 independent experiments, expressed as mean plus standard deviation. Data was analysed by one-way ANOVA with Tukey’s multiple comparison test and significance is shown as previously indicated

In order to analyze the relative transport of IgG1 and IgG2 and the role of FcγR and FcRn, we analyzed their transport *in vitro* using the choriocarcinomic JAR cells (endogenously expressing FcRn)^28^, A375 cells devoid of FcRn^28^ expression, and A375- FcRn transfectants^46^. None of these cell lines expressed classical FcγR as seen by FACS (Supplementary Fig. 1). As seen *in vivo*, we observed that IgG1 (B2G1) was transported more efficiently than IgG2 (B2G2) (an otherwise identical antibody of the IgG2 subclass) both by JAR cells (Fig. 2A) and A375-FcRn cells (Fig. 2B), but not FcRn-deficient wild type A375 cells (Fig. 2C).

### IgG transcytosis is independent of the ability to bind Fcγ receptors

We have previously described B2G1Δnac, a mutated IgG1 variant with amino acids which are implicated in FcγR binding being replaced by corresponding residues found in IgG2 or IgG4. This variant displays almost no binding to activating FcγR but retains 40% binding to the inhibitory FcγRIIb^39,47^. This mutant was transported with equal efficiency as wild type IgG1 antibody in JAR cells (Fig. 2A) and A375-FcRn cells (Fig. 2B). However, another variant, B2G1Δnab, identical and with similar FcγR-binding properties as B2G1Δnac but additionally lacking glycine 236 (G236) which is absent in wild type IgG2, was transported less efficiently and to the same extent as the wild type IgG2 molecule (B2G2) (Fig. 2A,B). This was observed in both JAR (Fig. 2A) and A375-FcRn cells (Fig. 2B) but no significant transport was found in wild type (WT)A375 cells lacking FcRn and FcγRs expression^28^ (Fig. 2C). These findings demonstrated that FcRn, and not classical FcγR, was required for transcytosis of IgG in both JAR and A375 cells and suggested that absence of G236 may reduce the efficiency of this transcytosis.

### Apical to basolateral transcytosis of IgG is influenced by glycine 236

**Figure 3:**
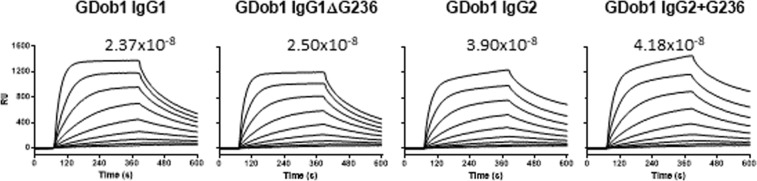
Mutant GDob1IgG1ΔG236 and GDob1IgG2 + G236 retain affinities to human FcRn in comparison to WT antibodies. Sensorgrams obtained from affinity measurement of GDob1IgG1, GDob1IgG1ΔG236, GDob1IgG2 and GDob1IgG2 + G236 to human FcRn in SPR. Antibodies were injected at concentrations ranging from 125 nM to 0.49 nM in two-fold dilutions over biotinylated human FcRn coupled to a streptavidin biosensor at pH 6.0. The affinities (M) calculated from affinity plots derived from SPR measurements of GDob1 IgG1, GDob1 IgG1 ΔG236, GDob1 IgG2 and GDob1 IgG2 + G236 to human FcRn. KDs were calculated using equilibrium analysis by intrapolation to Rmax = 1000 human FcRn. (−/−).

**Figure 4:**
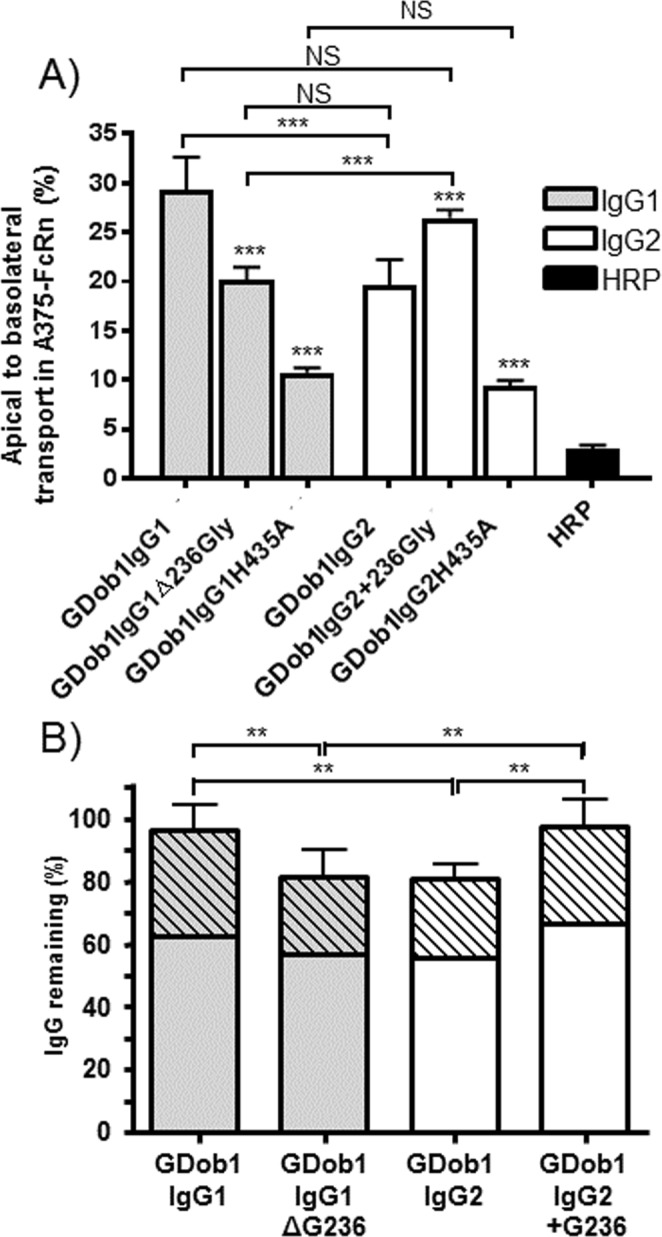
Low transport of IgG2 was due to lack of G236 in IgG2, and resulted in enhanced degradation. (**A**) Apical to basolateral transport of V_H_-matched GDob1 wild type and ΔG236 IgG1 variants as well as IgG2 wild type, and + G236 variants by A375 FcRn cells. GDob1IgG1H435A and GDob1IgG2H435A were included as control in which the main FcRn binding pocket was disrupted and HRP was included as a measure of passive diffusion. Experiments were run for 2 hours. (**B**) After 18 hours of apical to basolateral transport in A375-FcRn cells, both apical (hatched bars) and basolateral (open bars) compartments were sampled and IgG concentrations were determined. Approximately 95% of GDob1IgG1 and GDob1IgG2 + G236 could be accounted for, while only about 80% of GDob1IgG1Δ236Gly and GDob1IgG2 was detectable. Data shown are from three independent experiments, expressed as mean plus standard deviations. In B) the total values from apical and basolateral samples taken from the same transwells. Significance was tested by one way ANOVA with Tukeys multiple comparisons test in both (**A**,**B**). In (**A**) statistical comparison within an IgG subclass is shown without brackets and only shown for comparison with the WT variant, but between subclasses (with brackets) only shown for WT subclasses, G236 inserted in IgG2 and G236 removed from IgG1, and matched for the presence or absence of G236 IgG1 and IgG2 variants.

To ascertain whether the reduced IgG2 transport was due to the lack of G236 compared to IgG1, we generated recombinant antibodies differing only in this single amino acid. Affinity measurements by SPR with c-terminally biotinylated FcRn on streptavidin sensors showed that either removing G236 from IgG1 or introducing it into IgG2, did not apparently influence binding to FcRn on the chip (Fig. 3). The wild-type and mutant GDob1 antibodies displayed a similar minor fraction of dimers which was similar across all variants (Supplementary Fig. 2), also seen in rather steep associations and slow disassociation in Fig. 3. We found that IgG1 lacking only G236 (GDob1IgG1ΔG236) was transported to an equal level as wild type IgG2 (Fig. 4A). Moreover, IgG2 with G236 inserted (GDob1IgG2 + G236) demonstrated enhanced transport and was transported to an equal level as wild type IgG1, confirming that the lack of G236 in IgG resulted in its less efficient FcRn-mediated transcytosis. When the affinity of the primary binding site of IgG for FcRn was strongly reduced by changing the histidine in position 435 to an alanine (H435A)^48,49^ significant transport was still observed, but it was severely reduced as expected. Importantly, no significant difference between IgG1 and IgG2 transport was observed when both carried the H435A mutation, suggesting FcRn-mediated effector functions were responsible for the observed differences in IgG1 and IgG2 transport efficiency. In addition, role of FcγR can be excluded by their absence in these cells (Supplemental Fig. 1).

### Reduced FcRn mediated transport correlates with enhanced degradation

To investigate whether the reduced transcytosis rates of IgG without G236 were due to increased degradation, we sampled both apical and basolateral compartments after 18 hours of transport.

For both wild type GDob1IgG1 and GDob1IgG2 + G236 the total amounts detected were in excess of 95% of the starting material, while wild type GDob1IgG2 and GDob1IgG1ΔG236 were recoverable to a significantly lesser extent (Fig. 4B). This suggested that the reduced FcRn-mediated transcytosis of GDob1IgG2 and GDob1IgG1ΔG236 (Figs 1–2) was due to enhanced degradation.

## Discussion

The long half-life of IgG and its transplacental transport are both mediated by the neonatal Fc receptor^5,19,23^. FcRn-mediated half-life of IgG2 is comparable to that of IgG1^27^ but transport across the human placenta is lower for IgG2^21–26^, suggesting differences between the mechanisms of FcRn-mediated recycling and transcytosis of IgG. The reason for this is unclear, as IgG1 and IgG2 have not been reported to differ in residues found to be important for IgG-FcRn interaction^6^ and their binding to FcRn immobilized on a biosensor chip were not reported to be significantly different^31^. However, the stoichiometry^10^, the lateral fluidity of transmembrane receptors, and the physiological proximity of the plasma membrane cannot be accounted for on a surface like plasmon resonance biosensor chips, and therefore, such assays do not always accurately represent the biological context.

**Figure 5:**
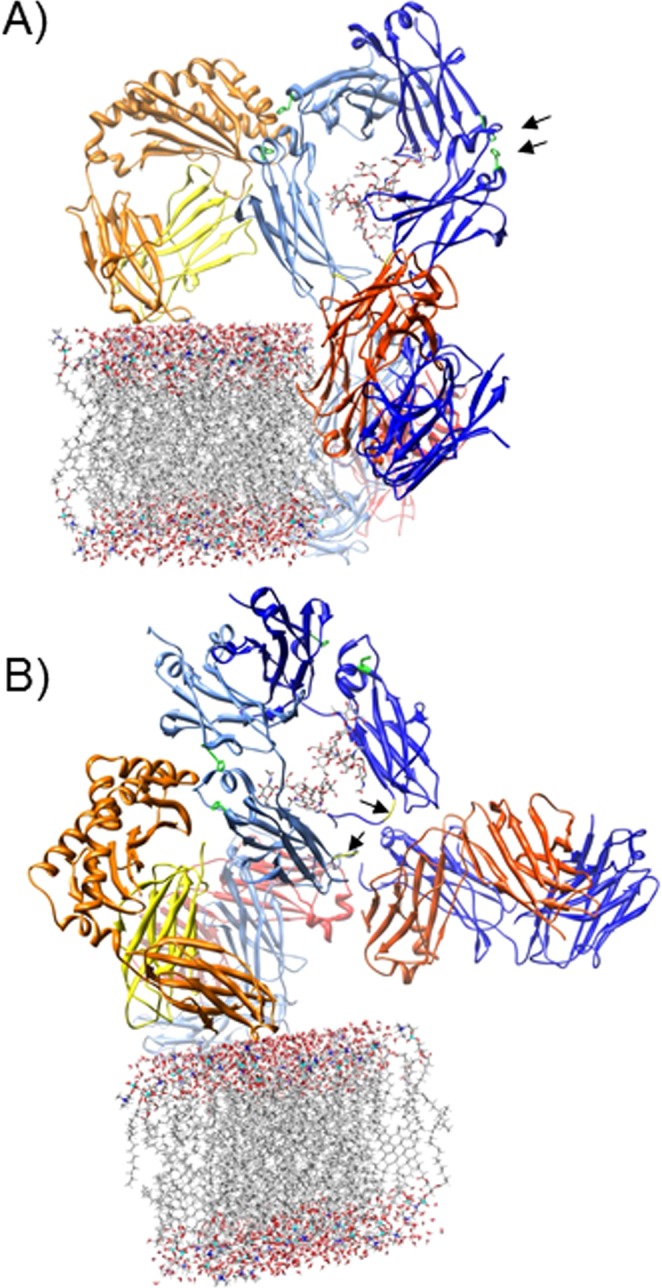
IgG binds FcRn in a top down orientation. IgG1 is depicted in red and blue colours (the two light chains in red and light red, and the two heavy chains in blue and light blue). The α-chain of FcRn is depicted in orange, and β2M in yellow and is modelled on a plasma membrane. The positions of the two potential FcRn binding sites on the IgG1 are indicated by showing the critical histidine residues in green and their position is indicated with arrows in (**A**) (upper arrow H435 in CH3; lower arrow H310 in CH2). One of the binding sites is unoccupied. The position of G236 is indicated in yellow and with arrows in (**B**). In (**A**), a model of FcRn binding to human IgG1 shows that the Fab portions of the molecule will clash into the plasma membrane if FcRn is extending directly from in the plasma membrane. This could be avoided if the Fab fragments bend back at the hinge and/or FcRn bends back against the plasma membrane, as in (**B**). Data are based on the crystal structure of rat FcRn with Fc (Accession number 1I1A Ref martin et al. mol cell 2001) and overlaid with the structure of human IgG1 (Accession number 1HZH, Saphire 2001 Science). UCSF Chimera (Pettersen, *et al*. 2004) was used for modelling and imaging.

The lowered *in vitro* FcRn-mediated transcytosis rates of IgG2 compared to IgG1 which we observed were in line with the reported differences in maternal-cord blood ratios. This difference was solely attributed to FcRn-mediated transcytosis as transcytosis of B2G1Δnac, an IgG1 variant unable to bind to classical FcγR^38,39,47^, was unaffected in both JAR and A375-FcRn cells. Unexpectedly, the transcytosis rate of another IgG1 variant, B2G1Δnab, was reduced to the level of IgG2 transcytosis. The only difference between the Δnac and Δnab variants was the deletion of G236 in the latter, similar to IgG2 that also lacks G236. Using unrelated human IgG1 and IgG2 V_H_-matched GDob1 antibodies^41,42^ with identical affinity for FcRn in SPR experiments, we confirmed that G236, but not any other mutations in B2G1Δnac or B2G1Δnab, influenced the transport of IgG. Interestingly, G236 has not previously been reported to affect FcRn function and is located in the lower hinge, far from the FcRn binding site^6^ (Fig. 5).

Gurbuxani *et al*. postulated a mathematical model for FcRn-IgG interaction^50,51^, in which either FcRn can bind to IgG in two distinct modes, or IgG might have two FcRn binding sites, functioning synergistically. Whether such a secondary interaction-domain within IgG exists is unclear, but it cannot be excluded as a cocrystal of a complete IgG-FcRn complex has not yet been resolved. Björkman and colleagues observed an FcRn dimer both in crystals of rat FcRn and in the cocrystal of FcRn with Fc^6,49^. Later work demonstrated that Fc and FcRn can crystallize in oligomeric ribbons with a symmetrical repeating unit of 2 FcRn:1 IgG (FcRn:IgG:FcRn) with the C-terminus of IgG orientated towards the N-terminus of FcRn^52^. Both the proposed “lying down” or “standing up” models for FcRn engaged with IgG binding have difficulties explaining how a complete IgG binds to FcRn in a cellular context. In the “standing up” model the two Fab fragments would be projected to either protrude into the plasma membrane, or, due to the flexibility of the hinge, bend back (Fig. 5A). The fragment antigen binding (Fab) might be more easily accommodated if FcRn is tilted towards the cell but some bending at the hinge would still be required (Fig. 5B). The binding of a second FcRn to this complex, forming a 2:1 complex of FcRn and IgG which might be important for FcRn-signalling events^16^, may occur within the IgG/FcRn positive-transport tubules observed by the groups of Ober and Ward *et al*.^53,54^. Within these tubules one molecule of IgG may be bound by two FcRn molecules residing in a tilted orientation on parallel membranes. This is similar to what was suggested to occur between adjacent microvilli in the rodent gut by Bjorkman *et al*.^6^, who observed by electron cryotomography that IgG-Fc complexes assemble preferentially on interfaces between layers of FcRn expressing membranes^54,55^. Whether this “laying down” model, the “standing up” model or both is more relevant for physical transport of IgG is currently unknown.

G236 is present in the lower hinge of all subclasses except IgG2, and increases the flexibility and extends the distance of the two Fab domains from the Fc portion. Based on the structural constraints for binding of whole IgG by FcRn, and the findings presented here, we predict that the shortened hinge of IgG2 makes the formation of an FcRn:IgG2:FcRn complex less energetically favourable than is the case for other IgG and thus decreases the compatibility of IgG2 with FcRn binding and transport. This is supported by recent evidence showing that the amino-acid composition of the Fab fragment and charge in particular effects FcRn binding and recycling^56^. Our study may in part provide a solution as to why IgG2 transcytosis takes place at a lower rate than IgG1. However, it does not explain why *in vivo* rates of IgG2 and IgG1 catabolism do not seem to differ, unless these two FcRn-mediated processes fundamentally differ, or hitherto unknown factors make IgG2 less prone to degradation, compensating for the reduced FcRn rescue function. This last option is however entirely speculative and we are not aware of any data indicating that this is the case.

In summary, we show that the lowered transplacental transport of IgG2 *in vivo* can be simulated in an *in vitro* transcytosis system, is FcRn- but not FcγR-mediated, and is caused by the absence of G236 in IgG2. More effort is needed to unravel the cellular mechanism behind the observation that the short lower hinge of IgG2 affects transcytosis but not its half-life.

## Supplementary information

Supplementary figure 1 and 2
