# Supplementary material for: Reduced FcRn-mediated transcytosis of IgG2 due to a missing Glycine in its lower hinge
Source: Sci Rep. 2019 May 14;9:7363. doi: 10.1038/s41598-019-40731-2 (PMC6517591; doi:10.1038/s41598-019-40731-2)
Supplement: Supplementary file 1 — Supplementary figure 1 and 2 [file 41598_2019_40731_MOESM1_ESM.pdf]

Supplementary data

Reduced FcRn-mediated transcytosis of IgG2 due to a missing Glycine in its lower hinge.

Nigel M. Stapleton [1,2], Maximilian Brinkhaus [1], Kathryn L. Armour [3,4,6], Arthur E.H. Bentlage [1], Steven W. de Taeye[1], A. Robin Temming[1], Juk Yee Mok[8], Giso Brasser[8], Marielle Maas[8], Wim J. E. van Esch[8], Mike R. Clark [3,7], Lorna M. Williamson [4,5], C. Ellen van der Schoot [1], Gestur Vidarsson [1*]

1: Sanquin Research, Department of Experimental Immunohematology, Amsterdam, The Netherlands, and Landsteiner Laboratory, Amsterdam UMC, University of Amsterdam, Amsterdam, The Netherlands, Plesmanlaan 125, Amsterdam 1066 CX, The Netherlands.
2: Current address: HALIX B.V., J.H. Oortweg 15 / 17, 2333 CH Leiden, The Netherlands
3: Department of Pathology, Division of Immunology, University of Cambridge, Tennis Court Road, Cambridge CB2 1QP, UK
4: Department of Haematology, University of Cambridge, UK
5: NHS Blood and Transplant, Long Road, Cambridge CB2 2PT, UK
6: Current address: LifeArc, Open Innovation Campus, Stevenage, SG1 2FX, UK
7: Current address: Clark Antibodies Ltd, 10 Wellington Street, Cambridge, CB1 1HW, UK
8: Sanquin Reagents, Amsterdam, Netherlands

Stapleton et al Supplemental Fig 1:

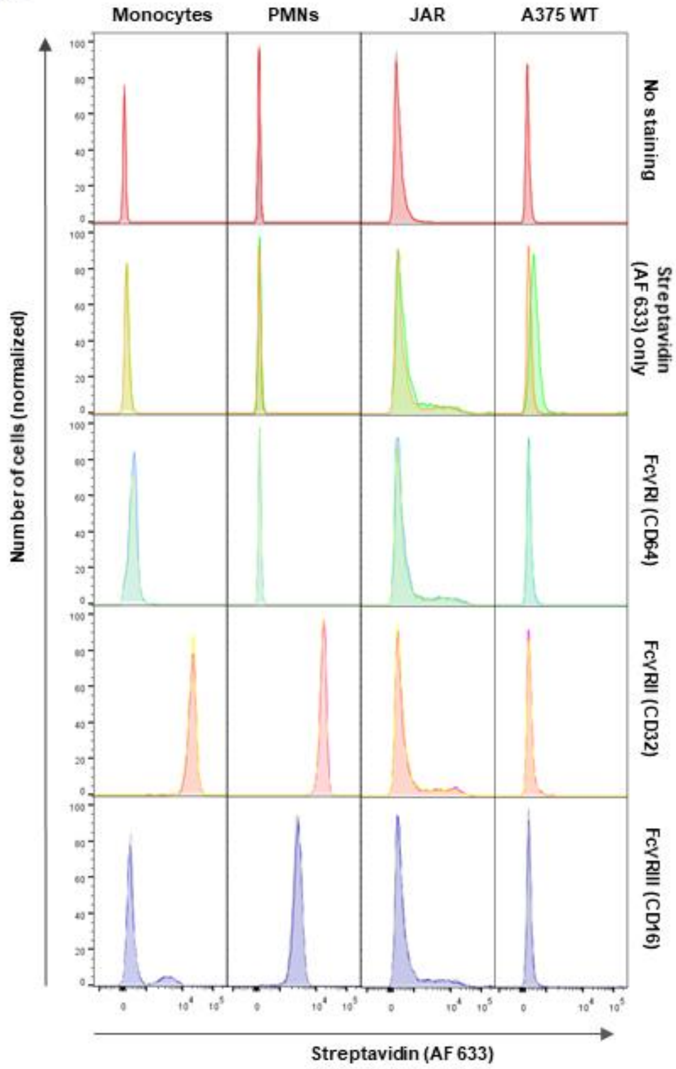

Supplementary figure 1: JAR and A375 WT cells do not express FcγRs, A375 FcRn cells show FcγRIIa expression.

Histograms from FACS analysis of FcγRI (CD64), FcγRII (CD32) and FcγRIII (CD16) expression levels on blood monocytes, polymorphonuclear leukocytes (PMNs), JAR-, A375 WT- and A375 FcRn cells. Plots are shown as ‘normalized to mode’ over AF 633 signal, duplicates are presented as overlay.

Stapleton et al Supplemental Fig 2:

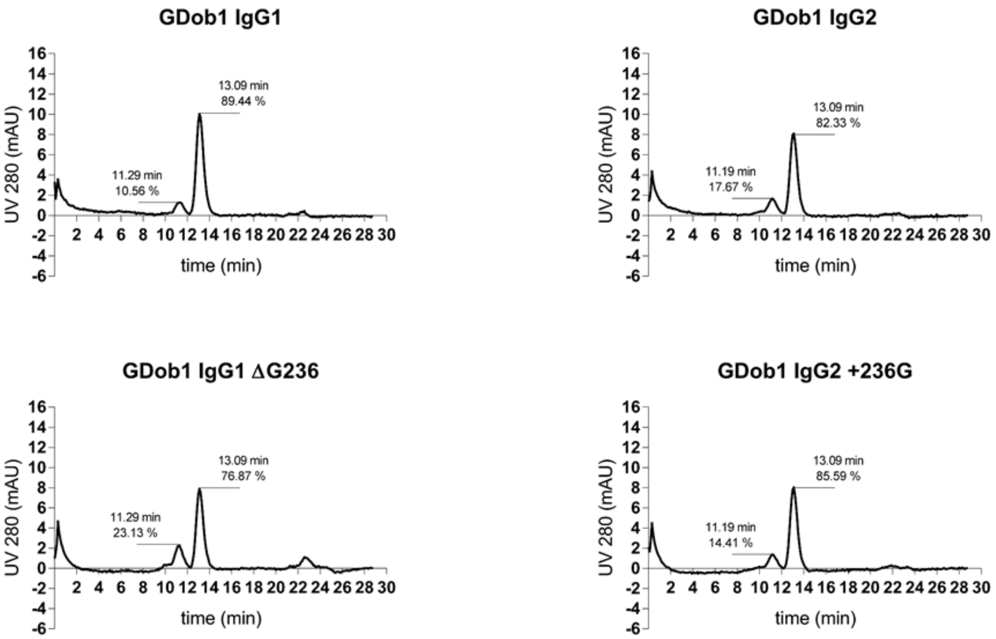

Supplemental Figure 2 : Mutants GDob1 IgG1 ΔG236 and GDob1 IgG2 +G236 do not show significant changes in levels of aggregation in comparison to WT antibodies. Aggregation levels of GDob1 IgG1, GDob1 IgG1ΔG236, GDob1 IgG2 and GDob1 IgG2+G236 were determined using HPLC-SEC. Results are shown as UV280 (mAU) over time (min). AUC values were calculated and Two-way ANOVA was used to determine statistical differences. Changes in levels of dimers and monomers were found to not be significant using Two-way ANOVA for statistical analysis.
